# Digest: A synergistic approach explains the evolutionary connection between brain size and longevity^*^

**DOI:** 10.1111/evo.14118

**Published:** 2020-11-10

**Authors:** Willian T.A.F. Silva

**Affiliations:** ^1^ Centre for Environmental and Climate Research Lund University Lund Sweden

## Abstract

The cognitive buffer hypothesis poses that brain size evolves to buffer individuals from environmental changes, increasing survival. Jiménez‐Ortega et al. (2020) explored this hypothesis using a phylogenetic path analysis and showed that there is a direct causal link between brain size and longevity in birds, even when allometric effects are taken into account. Furthermore, a synergistic model was better supported than models that included independent effects of brain size and body size.

Longevity may be one of the most important components of fitness because it can relate to the amount of time available for reproduction. The evolution of increased longevity can be a complex process that requires selection for mechanisms that help individuals cope with environmental changes. Examples of such mechanisms include the refinement of homeostatic control (Monaghan et al. 2009) and the development and enhancement of cognitive behavior, which allows individuals to actively minimize detrimental effects of environmental changes through behavioral adjustments (Allman 2000). Because of that, brain size is a primary candidate for selection to act upon to increase longevity.

According to the cognitive buffer hypothesis (CBH; Allman 2000), brain expansion evolves to buffer individuals against environmental variation. Although direct evidence supporting the CBH is poor, there is a clear positive correlation between brain size and longevity (González‐Lagos et al. 2010; Minias and Podlaszczuk 2017), which indicates the importance of brain size for survival.

In this issue, Jiménez‐Ortega et al. (2020) explore the CBH through the relationship between brain size and longevity in birds. They conducted a phylogenetic path analysis using data from across the avian phylogeny to test a variety of models that include different connections between brain size and longevity. Their analysis included both direct and indirect effects (Fig. 1A), taking into consideration life‐history traits and allometric effects in altricial bird species, which need parental care after hatching, and precocial bird species, which do not.

**Figure 1:**
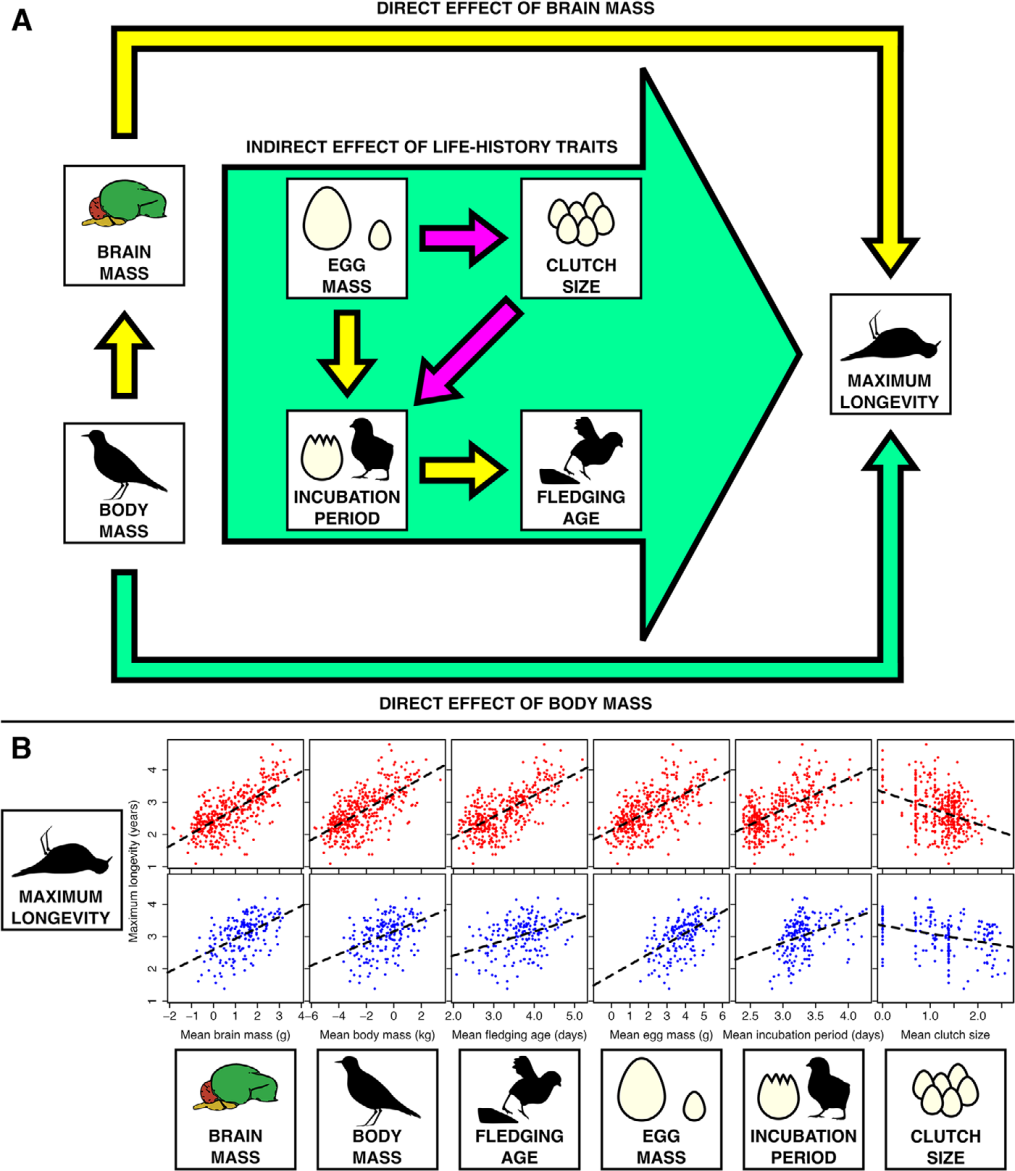
Brain and body mass can have direct or indirect effects (through life‐history traits) on maximum longevity. (A) Consistent connections and relationships between traits. Arrows indicate consensus positive (yellow), negative (pink), or mixed (green) relationships, according to average best‐supported models in altricial and precocial species. (B) Relationships between maximum longevity and several morphological and life‐history traits in altricial (red) and precocial (blue) species. Points represent log‐transformed mean values for different species. Dashed curves indicate the linear regression. Data from Jiménez‐Ortega et al. (2020).

Jiménez‐Ortega et al. (2020) found a strong direct relationship between brain size and longevity in birds, supporting the CBH. However, when different models were considered, those that assumed synergistic direct effects of brain size and body size on longevity in addition to indirect effects of life‐history traits (Fig. 1A) were better supported than models that assumed independent effects of brain size and body size. This finding indicates that synergistic approaches are particularly important for determining the functional connection between brain size and longevity.

Although results were qualitatively consistent in altricial and precocial species, differences between developmental modes emerged. For example, fledging age was particularly explanatory in altricial species, but not in precocial species; on the other hand, clutch size and egg mass were particularly explanatory in precocial species, but not in altricial species. Despite these differences, the direction of the independent effects of life‐history traits on longevity were consistent between developmental modes (Fig. 1B), indicating that developmental mode is not a strong explanatory factor when it comes to the connection between brain size and longevity.

Jiménez‐Ortega et al. (2020) shed light on the causal link between brain size and longevity, supporting the CBH. However, the exact functional mechanism through which brain size affects longevity may be lineage specific, with different environmental pressures selecting for different cognition traits and, therefore, enhancement of different brain regions. Because of that, future analyses of brain composition in different lineages may indicate the types of functional enhancement that have evolved across the phylogeny.

## CONFLICT OF INTEREST

The author declares that there is no conflict of interest.

Associate Editor: K. Moore

Handling Editor: T. Chapman

## SUBMIT A DIGEST

Digests are short (∼500 word), news articles about selected original research included in the journal, written by students or postdocs. These digests are published online and linked to their corresponding original research articles. For instructions on Digests preparation and submission, please visit the following link: https://sites.duke.edu/evodigests/
